# Emphysematous cholecystitis in a patient with porcelain gallbladder

**DOI:** 10.1093/jscr/rjac600

**Published:** 2023-01-17

**Authors:** Vithya Vera, Abhimanyu Sharma, Adam T Townson, Qasim Azeem, Yogeshkumar Malam, John Mathews

**Affiliations:** Department of Surgery, Hinchingbrooke Hospital, Huntingdon, UK; Department of Surgery, Hinchingbrooke Hospital, Huntingdon, UK; Department of Surgery, Hinchingbrooke Hospital, Huntingdon, UK; Department of Surgery, Hinchingbrooke Hospital, Huntingdon, UK; Department of Surgery, Hinchingbrooke Hospital, Huntingdon, UK; Department of Surgery, Hinchingbrooke Hospital, Huntingdon, UK

## Abstract

Our case uniquely presents a patient with two rare gallbladder disease entities occurring simultaneously. The patient presented to hospital with abdominal pain and was subsequently diagnosed with emphysematous cholecystitis and porcelain gallbladder. After initial conservative management failed, cholecystectomy was performed, and the patient recovered well post-operatively and was discharged home.

## INTRODUCTION

Acute cholecystitis is a common surgical condition characterized by right upper quadrant abdominal pain and raised inflammatory markers. Although most cases resolve with antibiotic treatment, emphysematous cholecystitis is a rare, severe manifestation of acute cholecystitis and is associated with increased morbidity and mortality. In this report, we describe a patient with concurrent porcelain gallbladder and emphysematous cholecystitis and their management. Simultaneous occurrence of these two rare entities has not previously been described.

## CASE PRESENTATION

A 57-year-old woman presented to the emergency department with a 1-week history of right upper quadrant abdominal pain and nausea. The patient denied any fevers or jaundice.

Past medical history was significant for type 2 diabetes mellitus, hypertension and depression. The patient’s medications included metformin, gliclazide, atorvastatin, sertraline and propranolol.

Vital signs on admission revealed a blood pressure of 135/58 mmHg, pulse rate of 72 beats per minute and temperature of 36.8 °C. Clinical examination revealed a tender palpable mass in the right upper quadrant of the abdomen with no signs of peritonism.

**Figure 1:**
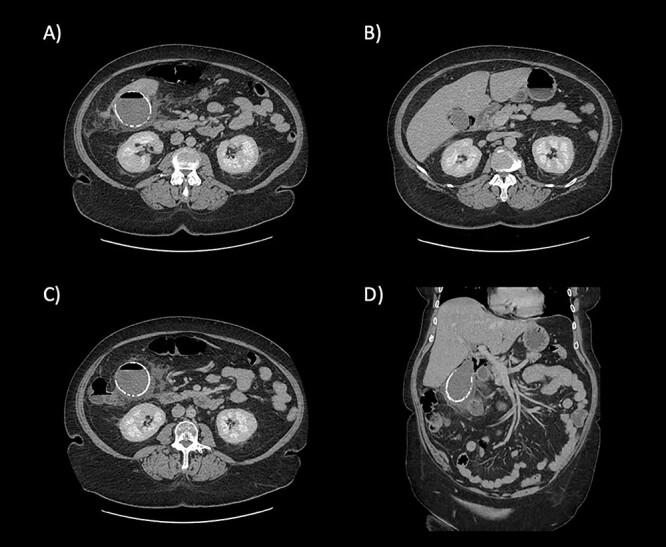
CT scan images demonstrating the calcified wall of the gallbladder consistent with a porcelain gallbladder **(A)**. Pericholecystic fluid and stranding (**A** and **B**) demonstrate cholecystitis. The presence of air within the wall and lumen of gallbladder is consistent with emphysematous cholecystitis (**C**). Air is also seen tracking along the hepatic hilum **(D)**.

Blood tests on admission showed raised inflammatory markers (white cell count 11.3 × 10^9^ /L; C-reactive protein 78 mg/L) and raised bilirubin 37 µmol/L. Alanine aminotransferase, alkaline phosphatase and renal function were all within normal limits. A contrast-enhanced computer tomography (CT) scan of the abdomen and pelvis demonstrated a calcified porcelain gallbladder and with features of emphysematous cholecystitis (Fig. 1).

Intravenous antibiotics (piperacillin with tazobactam) were commenced, and the patient initially managed conservatively. However, by day 3 of admission, the patient failed to improve clinically, and the decision was made to proceed to laparoscopic cholecystectomy.

**Figure 2:**
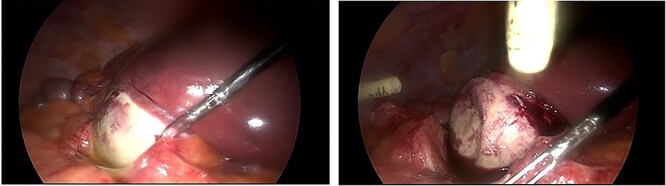
Intraoperative images demonstrating an inflamed porcelain gallbladder.

Laparoscopy revealed an acutely inflamed porcelain gallbladder (Fig. 2). Laparoscopic dissection of Calot’s triangle was difficult due to inflammation and the decision was made to convert to an open operation to complete the cholecystectomy safely. A fundus first approach was employed and the cystic duct was found to be obliterated by an impacting gallstone. To minimise the risk of common bile duct injury, the decision was made against further dissection of the cystic duct stump. The cholecystectomy was completed, and a drain was left in the subhepatic space.

The post-operative recovery was complicated by a perihepatic collection, which was successfully managed conservatively with intravenous antibiotics. The patient was discharged on Day 7 post-operatively.

**Figure 3:**
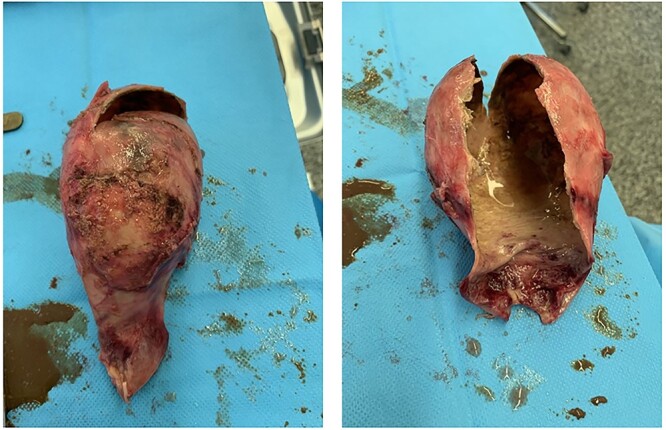
Surgical specimen demonstrating the calcified brittle wall of the porcelain gallbladder.

Histological examination of the resected gallbladder demonstrated features of inflammation with dystrophic calcification of the gallbladder wall (Fig. 3). No evidence of gallbladder dysplasia or malignancy was seen.

## DISCUSSION

Porcelain gallbladder is a rare clinical entity that arises from calcification of the gallbladder wall. Most patients are diagnosed incidentally on imaging, where the pathognomonic appearance of a radiopaque porcelain gallbladder is seen on X-rays and CT. The underlying etiology is uncertain, but porcelain gallbladder is usually associated with the presence of gallstones.

Early literature suggested an increased risk of gallbladder cancer in patients with porcelain gallbladder, and prophylactic cholecystectomy was therefore traditionally performed in these patients. However, more recent evidence disputes the strength of this association [1–3]. The association between porcelain gallbladder and gallbladder malignancy is not as strong as previously thought, and some argue that prophylactic cholecystectomy is no longer mandated.

Emphysematous cholecystitis is a rare form of acute cholecystitis characterized by the presence of gas in the gallbladder lumen, wall or pericholecystic tissues (without the presence of a communication between the biliary system and the gastrointestinal tract). It is mainly caused by gas-forming bacteria but may also be associated with vascular insufficiency or ischemia of gallbladder due to endarteritis obliterans.

Emphysematous cholecystitis is commonly associated with advanced age, frailty and poor diabetic control [4]. It is associated with increased rates of gallbladder perforation and caries mortality rates ~25%.

Our case uniquely describes the presentation of two rare gallbladder disease entities occurring simultaneously. Though technically challenging, cholecystectomy was successfully performed and the patient recovered well post-operatively, highlighting the importance of early decision making in challenging cases of complicated cholecystitis.

## FUNDING

None.

## CONFLICT OF INTEREST STATEMENT

None declared.

## DATA AVAILABILITY

The data underlying this article will be shared on reasonable request to the corresponding author.
